# The Impact of Rainfall on Soil Moisture Dynamics in a Foggy Desert

**DOI:** 10.1371/journal.pone.0164982

**Published:** 2016-10-20

**Authors:** Bonan Li, Lixin Wang, Kudzai F. Kaseke, Lin Li, Mary K. Seely

**Affiliations:** 1 Department of Earth Sciences, Indiana University-Purdue University Indianapolis (IUPUI), Indianapolis, IN 46202, United States of America; 2 Desert Research Foundation of Namibia, Windhoek, Namibia; Tennessee State University, UNITED STATES

## Abstract

Soil moisture is a key variable in dryland ecosystems since it determines the occurrence and duration of vegetation water stress and affects the development of weather patterns including rainfall. However, the lack of ground observations of soil moisture and rainfall dynamics in many drylands has long been a major obstacle in understanding ecohydrological processes in these ecosystems. It is also uncertain to what extent rainfall controls soil moisture dynamics in fog dominated dryland systems. To this end, in this study, twelve to nineteen months’ continuous daily records of rainfall and soil moisture (from January 2014 to August 2015) obtained from three sites (one sand dune site and two gravel plain sites) in the Namib Desert are reported. A process-based model simulating the stochastic soil moisture dynamics in water-limited systems was used to study the relationships between soil moisture and rainfall dynamics. Model sensitivity in response to different soil and vegetation parameters under diverse soil textures was also investigated. Our field observations showed that surface soil moisture dynamics generally follow rainfall patterns at the two gravel plain sites, whereas soil moisture dynamics in the sand dune site did not show a significant relationship with rainfall pattern. The modeling results suggested that most of the soil moisture dynamics can be simulated except the daily fluctuations, which may require a modification of the model structure to include non-rainfall components. Sensitivity analyses suggested that soil hygroscopic point (*s*_h_) and field capacity (*s*_fc_) were two main parameters controlling soil moisture output, though permanent wilting point (*s*_w_) was also very sensitive under the parameter setting of sand dune (Gobabeb) and gravel plain (Kleinberg). Overall, the modeling results were not sensitive to the parameters in non-bounded group (e.g., soil hydraulic conductivity (*K*_s_) and soil porosity (*n*)). Field observations, stochastic modeling results as well as sensitivity analyses provide soil moisture baseline information for future monitoring and the prediction of soil moisture patterns in the Namib Desert.

## 1. Introduction

It has long been suggested that soil moisture is a critical component of earth systems [1]. Although the amount of soil moisture is relatively small when compared with other constituents of the hydrological cycle [2, 3], it is of great importance to many hydrological, biological and biogeochemical processes. Soil moisture, as one source of water for the atmosphere through evapotranspiration [4], is a key variable in controlling the exchange of heat fluxes between the land surface and the planetary boundary layer [5]. Spatial and temporal variability of soil moisture provides an essential indicator for evaluating and understanding vegetation patterns and dynamics [6, 7]. Soil moisture also controls microbial dynamics and affects a number of soil chemical/physical properties [8], such as O_2_ levels, pH and the concentration of mineral nutrients (e.g., ferric iron) in soil solution, which in turn affect activities and population dynamics of microbial biomass.

Soil moisture is especially important to link climate, soil, and vegetation in dryland ecosystems. Drylands cover about 40% of the earth surface and are typically located in continental regions where rainfall is less than potential evapotranspiration [9–11]. Dryland soil water content is typically low, but soil water is critical for vegetation dynamics and moisture in the topsoil can effectively protect the dryland soil from wind erosion [12, 13]. Typically, precipitation is the major source of soil moisture in drylands, though in some fog dominated systems, the non-rainfall water (e.g., fog and dew) can exceed annual rainfall [14]. Dryland near-surface climate is affected by soil moisture, which has been revealed as a major factor contributing to the occurrence of extremely high temperature and drought weather [10]. Therefore, understanding interactions between soil moisture and precipitation is critical to predict the response of dryland ecosystems to global environmental changes. To make a long-term prediction of soil moisture, process-based modeling is often required. One of the recent advances in ecohydrology is the successful modeling of stochastic rainfall-soil moisture relationships. Recently a modeling framework was developed based on the stochastic characteristic of rainfall events and analytical results of the probability distributions of soil moisture were successfully obtained. The modeling framework was improved and modified in a previous study to achieve a more accurate description of soil moisture dynamics especially in water-limited systems [15]. A series of studies have applied the modeling framework to different dryland environments [16–18]. Although the modeling framework has been used for years, the sensitivities of the key parameters and their dependence on soil texture are poorly understood. The hyper-arid environment of the Namib Desert and the diverse soil textures under similar rainfall regimes make it an ideal place to test the sensitivities across various soil textures.

The Namib Desert, which is one of the oldest and largest deserts, is located between a highland plateau and the Atlantic Ocean [19]. The hyper-arid environment of the Namib Desert was formed by the cold subantarctic upwelling combined with a hot subtropical interior, resulting in a bleak coastal condition [20, 21]. The annual average rainfall of the Namib Desert is typically low and the distribution is very heterogeneous. The western Namib Desert, on average receives about 5 mm annual rainfall while the eastern part receives about 85 mm [22, 23]. In addition to the hyper-arid environment and extremely rare rainfall, the frequent occurrence of fog is the most distinctive characteristic in the Namib Desert [21]. The fog in the Namib Desert is often considered as a westerly advection fog mainly driven by the Bengula cold current [24]. It has long been observed that the Namib Desert fog forms from the coastal areas between midnight and morning and dissipates towards noon. After fog forms at the coastal areas, it is pushed inland by the westerly wind resulting in a west-east gradient foggy zone [25]. The Namib Desert fog as a source of water has been playing an important role in sustaining plant growth by means of interception and can also be used for the survival of small animals [26, 27]. For example, fog water uptake has been observed for three beetle species of the Namib Desert when the beetles face extreme surface temperature and wind [28]. The Namib grass *Stipagrostis sabulicola* was found to rely heavily on fog water to sustain themselves and is able to transfer fog water intercept by leaves to their plant base by stem flow [29]. It has also been reported that 19% of the water within the *Sequoia sempervirens*, and 66% of the water within the understory plants come from fog in the California redwood forests [30, 31].

Despite the recognition of the importance of soil moisture in controlling various ecohydrological processes in the Namib Desert, soil moisture dynamics and how much soil moisture variability can be explained by rainfall in the foggy Namib Desert have not been reported. In this study, twelve to nineteen months’ daily records of rainfall and soil moisture records from diverse ecosystems in the Namib Desert were reported. The objects of this study are to, 1) present field observations of soil moisture and rainfall records acquired from different soil types in the Namib Desert; 2) use process-based modeling to simulate soil moisture dynamics under diverse soil textures; and 3) quantify the sensitivity of the stochastic modeling with a range of soil and vegetation parameters.

## 2. Materials and Methods

### 2.1 Field sites

**Fig 1:**
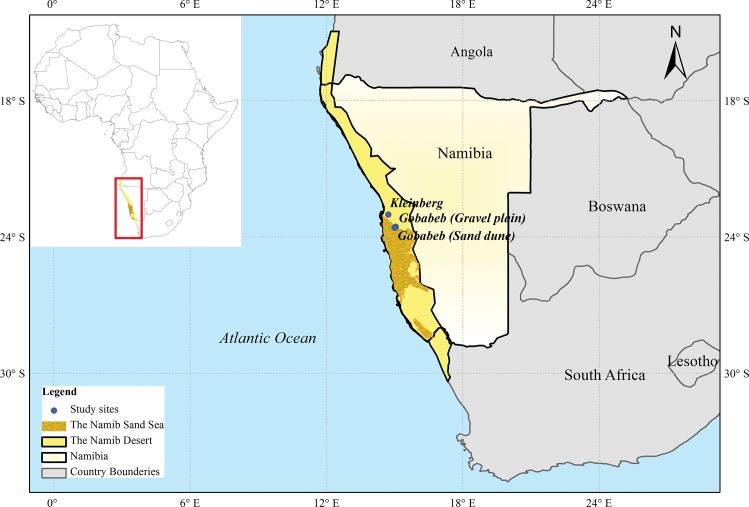
Location of the Namib Desert and the Namib “Sand Sea”. Blue points show locations of sites in Gobabeb and Kleinberg. The map was generated using ArcGIS for Desktop 10.3.1 (http://www.arcgis.com).

The Namib Desert is an ancient desert located in the coastal area of Namibia. It has a total length of 1900 km along the coast of the Atlantic Ocean, from the Olifants River in South Africa to Carunjamba River in Angola (Fig 1). The average rainfall in the Namib Desert ranges from 50 to 100 mm in the far south, 5–18 mm in the central Namib Desert and less than 50 mm along the Angolan coast in the north [22, 26]. Three main land forms are found in the Namib Desert. The southern Namib Desert is mainly covered by the endless sand dunes called the Namib “Sand Sea”, whereas gravel plains are dominant in the Central Namib dotted with inselbergs of granite and limestone. Moving north from the central Namib Desert, gravels plains finally give way to rugged mountains and dune fields [32].

Gobabeb is located at the edge of the Namib “Sand Sea” (Fig 1), 60 km inland from the Atlantic Ocean [22]. The climate is hyper-arid with average annual rainfall less than 50 mm primarily concentrated around January to March. Two different landscapes occur near Gobabeb along both sides of the Kuiseb River, the “Sand Sea” to the south and the gravel plains (calcrete soil) to the north [33]. Major plant species in the gravel plain are *Zygophyllum simplex* and *Z*. *stapffi*, while *Stipagrostis sabulicola* and *Trianthema heroensis* are the most commonly seen species dotted in the sand dune area [34]. Kleinberg (Fig 1), located about 33 km from the Atlantic Ocean, has been a field site attached to the Gobabeb Research and Training Centre for over 30 years, with most of the area covered by gravel plains (gypsum soil) [33] dotted with pencil bush (*Arthraerua leubnitziae*) and assorted lichens. The total annual rainfall amounts in both Gobabeb and Kleinberg are similar and rainfall events rarely occur.

### 2.2 Data collection

Three sites (Kleinberg gravel plain, here after GPK; Gobabeb sand dune (High Dune), here after SDG; Gobabeb gravel plain, here after GPG) were selected in our study because of the similar meteorological conditions and different soil textures among these three sites. Twelve to nineteen months’ volumetric soil moisture data and the corresponding rainfall data (January 1, 2014 to August 3, 2015 for GPK; July 28, 2014 to July 28, 2015 for SDG; January 2, 2014 to July 28, 2015 for GPG) were collected and used to test the stochastic modeling (data were available in S1 Table). The data collection was granted with a research permit from Gobabeb Research and Training Center of Namibia. Daily rainfall data were obtained from two tipping-buckets (one at GPG and another at GPK), which have been calibrated in the field. The same tipping bucket data was used for GPG and SDG because of their proximity (about 3.5 km apart). Soil moisture from both bare soil and vegetated areas were continuously monitored at one-hour interval using CS655 Water Content Reflectometer (Campbell Scientific, Inc. Logan, Utah, USA). In total six probes were used to measure soil moisture under different layers at the three sites. Three probes were used at GPG with two being installed at the same location under bare soil at 7.5 cm and 22.5 cm and the other one being installed under vegetation cover at 7.5 cm. Two probes were installed at SDG with one under bare soil at 15 cm and another one under vegetation cover at 7.5 cm. The depths of the two probes at the sand dune site were different due to the movement of shifting sand. One probe was installed at GPK under bare soil at 5 cm. Saturated hydraulic conductivity (*K*_s_) were estimated using the mini disk tension infiltrometer (Decagon Inc. Pullman, WA, USA) from multiple locations at each site.

### 2.3 Data analyses of the field data

Hourly volumetric soil moisture data were averaged to daily scale in order to match the model time scale. Data central tendency and variability were reported as mean, standard deviation, and coefficient of variation. The Mann–Whitney U test was deployed to examine the differences of mean soil moisture among the three sites in PAST (Paleontological Statistics, Natural History Museum, University of Oslo). Surface soil moisture distribution was examined using Q-Q/P-P plot in IBM SPSS (IBM Inc. NY, USA) and the corresponding probability density functions (pdfs) were described as Gamma distributions using two parameters (shape parameter *k* and scale parameter θ) in MATLAB. Correlations between rainfall events and soil moisture were tested using Pearson’s correlation. The significance level was set as α = 0.05.

### 2.4 Model structure and parameterization

Field soil moisture observations were modeled at daily scale by utilizing a process-based stochastic model. The model was defined with rainfall inputs as a non-homogeneous Poisson process with storm arrival rate λ and mean event depth α. However, in this study, we used a deterministic approach using field rainfall data to drive the model. Soil moisture dynamics are expressed as following equations:

$$nZ_{r}\frac{ds}{dt}=I(t,\;s)-ET(s)-L(s),$$ (1)

where *n* is the porosity, *s* is the relative soil moisture, *Z*_*r*_ is active soil depth or rooting depth, *I*(s, t) is the infiltration rate of rainfall, *ET*(s) is the rate of evapotranspiration, and *L*(s) is the rate of leakage or the loss of soil moisture from the bottom layer. The model assumes that all the water input from rainfall is immediately infiltrated into the ground and no surface runoff is generated.

There are three major factors in Eq (1) controlling soil moisture content. The increase of soil moisture is due to infiltration and the decrease of soil moisture is due to evapotranspiration and leakage through the bottom layer. The combined effect of those processes can be described as:

$$L(s)+ET(s)=\left\{ \begin{array}{l} E_{vap}+(E_{max}-E_{vap})\frac{s^{*}-s}{s^{*}-s_{w}}\;s_{w}<s\leq s^{*}\\ E_{max}\;s^{*}<s\leq s_{fc}\\ E_{max}+\frac{K_{s}}{e^{\beta (1-s_{fc})}}(e^{\beta (1-s_{fc})}-1)\;s_{fc}<s\leq 1 \end{array}\right.,$$ (2)

where *s*_w_ is the soil water content at wilting point, *s*_fc_ is the soil water content at field capacity, s* is the soil moisture in conditions of incipient stress, *K*_s_ is the hydraulic conductivity, *β* = 2*b* + 4, with *b* being the pore size distribution index; *E*_vap_ is the evaporation rate of ground surface while *E*_max_ being the maximum evapotranspiration under well-watered condition. For *s* > *s*_*fc*_, losses of soil moisture come from evapotranspiration and leakage. For s* < *s* ≤ *s*_fc_, only evapotranspiration contributes to the loss of soil water at the maximum evapotranspiration rate *E*_max_. For *s*_w_ < *s* ≤ s*, vegetation begins to suffer from water stress and regulates the transpiration rate through stomata closure. Thus, transpiration starts to be limited by soil moisture and the total evapotranspiration also decreases with decreasing soil moisture. For *s* < *s*_w_, *ET*(s) linearly decreases reaching a zero at *s* = *s*_h_ (hygroscopic point) where soil begins to absorb water from the atmosphere.

### 2.5 Model sensitivity analyses

Sensitivity analyses were conducted to examine the response of the modeled soil moisture output to soil and vegetation parameters under different soil textures. Sensitivity analyses were conducted by changing one parameter while fixing others (i.e., no interactive effects were tested). Before sensitivity analyses, we divided the key model parameters into bounded group (e.g., porosity (*n*), field capacity (*s*_fc_)) and non-bounded group (soil depth (*Z*_r_), saturated hydraulic conductivity (*K*_s_)) in terms of whether they have been normalized to the range of 0 to 1 or not. The maximum values of the bounded group were all set to 1, while the maximum values of the non-bounded group were set to different values. We predefined 20 as the maximum value for *Z*_*r*_, *T*_max_ and *E*_w_. Meanwhile, 100 was set to be the maximum value for *K*_s_ considering its magnitude in reality. Then in order to ensure the accuracy and precision of our sensitivity analyses, the parameter ranges were further refined to determine new parameter ranges based on the model outputs obtained from the previous procedures. Finally, based on the curve shape of the model output, we divided them into three groups: (1) monotonic increasing group, (2) monotonic decreasing group, and (3) non-monotonic group. For monotonic groups, the minimum values of the parameter ranges were defined as values that start making average-simulated soil moisture greater than zero while the maximum values were defined as values after which the average-simulated soil moisture will level off within the predefined or normalized ranges. For the non-monotonic group, the largest monotonic range (either monotonic increasing or decreasing) within the predefined or normalized range was regarded as the final range of the parameter. The average of the difference between two consecutive soil moisture output values divided by the parameter increment was then define as the parameter’s sensitivity, which can be described as:

$$Sensitivity=\frac{\sum_{i=2}^{n}\left(s(i)-s(i-1)\right)}{(n-1)*x}*100\%,$$ (3)

where *s* (i) is the correspondent soil moisture value, *s* (i-1) is soil moisture value one increment before *s* (i) produced within the predefined parameter range, *n* is the number of values, which equals to the determined parameter range divided by the parameter increment, *x* is parameter increments (e.g., 0.001 for porosity).

## 3. Results and Discussion

### 3.1 Field observations

**Fig 2:**
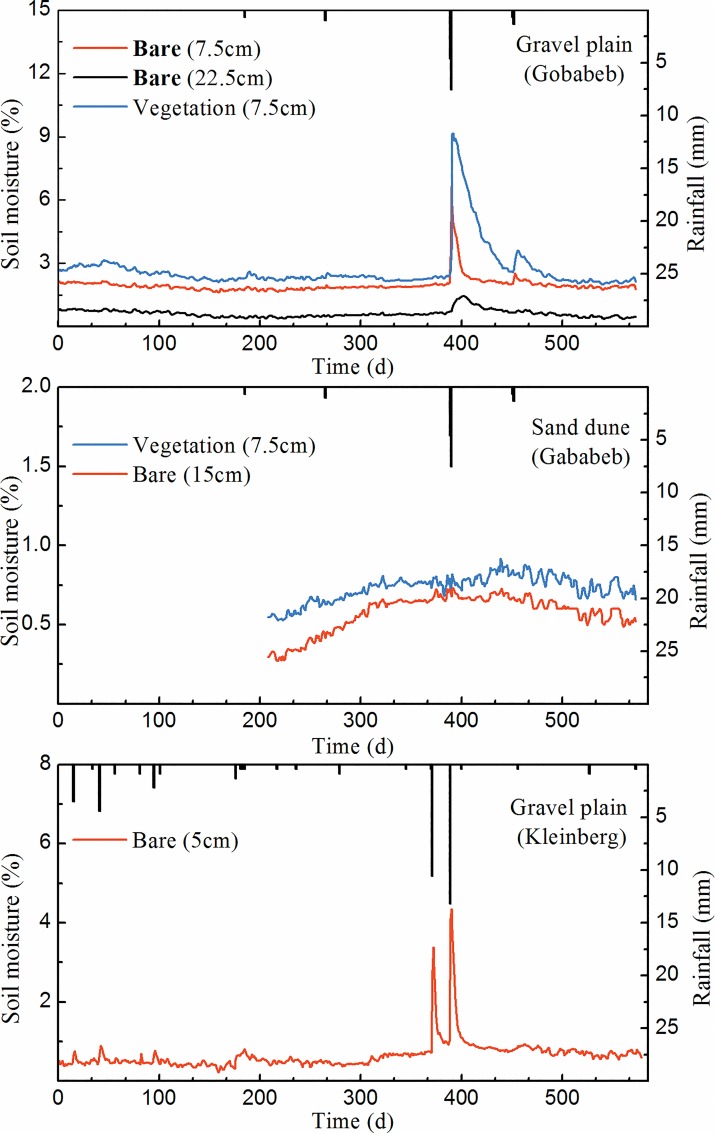
Rainfall regimes and volumetric soil moisture patterns for different depth of soil types in gravel plain at Gobabeb (GPG), sand dune at Gobabeb (SDG) and gravel plain at Kleinberg (GPK).

Table 1 shows the rainfall parameters and mean soil moisture values measured at the three sites. It was observed that total rainfall at GPG and SDG was 15.75 mm in 2014, which was slightly less than that at GPK (18.45 mm, Table 1). Despite average rainfall depth for GPG/SDG (2.63 mm) was slightly higher than that of GPK (1.95 mm), rainfall frequency at GPG/SDG (0.01) was only one quarter of that in GPK (0.04) (Table 1). Although divergences of rainfall parameters and total annual rainfall amount existed among these sites, the rainfall patterns were generally the same (Fig 2). Most of the rainfall concentrated on the wet season from November to May and large rainfall events mainly occurred in January (Fig 2).

**Table 1:** Mean soil moisture, standard deviation, coefficient of variation (CV), rainfall depth (mm), rainfall frequency λ (unitless) and average rainfall depth α (mm) for different soil depths of Gravel plain (Gobabeb) (January 2, 2014 to July 28, 2015), Sand dune (Gobabeb) (July 28, 2014 to July 28, 2015) and Gravel plain (Kleinberg) (January 1, 2014 to August 3, 2015).

Study site	Bare soil/Vegetation	Mean soil moisture (%)	CV (%)	Rainfall (mm)	λ	α (mm)
Gravel plain (Gobabeb)	***Bare soil (7*.*5cm)***	1.97±0.39	19.79	15.75	0.01	2.63
	***Bare soil (22*.*5cm)***	0.62±0.19	30.65	15.75	0.01	2.63
	Vegetation (7.5cm)	2.69±1.07	39.78	15.75	0.01	2.63
Sand dune (Gobabeb)	Bare soil (15cm)	0.57±0.12	21.05	15.75	0.01	2.63
	Vegetation (7.5cm)	0.73±0.08	10.96	15.75	0.01	2.63
Gravel plain (Kleinberg)	Bare soil (5.0cm)	0.70±0.40	57.14	18.45	0.04	1.95

Note: The bold letters refer to two sensors at different depths of the same location.

**Fig 3:**
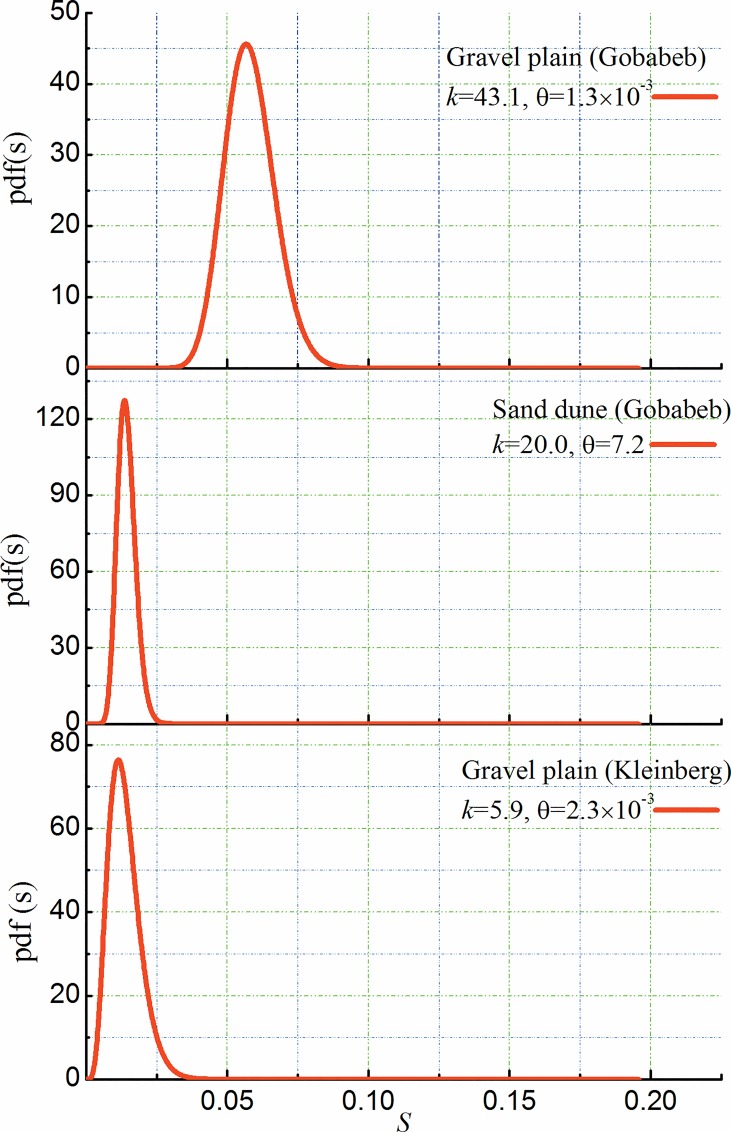
Relative soil moisture probability density functions (pdfs) for gravel plain at Gobabeb (GPG), sand dune at Gobabeb (SDG) and gravel plain at Kleinberg (GPK).

Fig 3 shows the simulated soil moisture probability density functions (pdfs) of GPG (bare soil 7.5 cm), GPK (bare soil 5 cm) and SDG (bare soil 15 cm) based on field measurements. Soil moisture pdf shape of GPG was different from that of SDG, which had smaller mode value and longer tail. The difference can be directly reflected by the values of shape parameter *k* and scale parameter *θ*, with GPG having a higher *k* (43.1) and lower *θ* (1.3 x 10^−3^). The discrepancy of soil moisture pdfs between GPG and SDG may result from the different antecedent soil moisture and the similarity of rainfall patterns (Fig 2). Soil moisture pdfs of SDG and GPK shared some similarities, with relatively low *k* (20.0 and 5.9, respectively) and high *θ* (7.2 and 2.3 x 10^−3^, respectively). The similarities may be explained by the proximity of their initial soil moisture of SDG and GPK and the resemblance of soil moisture mode value (Fig 2).

Significant differences of soil moisture dynamics can be found at different depths of soil layers at GPG (Fig 2) and soil moisture differences became larger after storms. Soil moisture difference between layers might be explained by the high dependence of surface soil moisture on the prevailing environment since it is continuously gaining and loosing water by means of rainfall infiltration and evapotranspiration. The phenomenon is more pronounced in the unsaturated zone or drylands where soil moisture largely depends on meteorological conditions [35, 36]. This behavior is also consistent with observations from other studies in arid regions [37, 38] but differs from the results obtained in China where researchers found no significant differences for soil water content at 0.4 m, 0.6 m and 0.8 m in a small watershed [39]. Besides, soil moisture differences between different layers were even found to be smaller after storms [40, 41]. The consistency between our results and previous research may be explained by the similarities of meteorological conditions. In general, low and irregular annual rainfall tend to result in high soil moisture in the surface soil and low soil moisture in the deep soil layer because when rainfall amount is too small, most of them will be retained in the shallow layer. That may help explain why soil moisture differences between surface layer and deeper layer tend to be larger after storms in our study sites. The discrepancy between our results and other studies may be induced by the difference in soil texture and hydrological conditions. Soils in other study sites may be able to hold less moisture, or have higher infiltrate rate or have more intense interaction with the groundwater, which may result in insignificant differences between shallower and deeper layers event after a strong storm. Soil moisture under vegetation cover was higher than that of bare soil at GPG (p > 0.05) (Fig 2, Table 1). This phenomenon is also suggested by other dryland studies [42]. This might be explained by the fact that bare soils tend to dry out faster due to the higher solar energy they receive. In addition, the net gain between retaining infiltrating rainfall and soil moisture losses through evapotranspiration under vegetated soil is likely larger than bare soil moisture loss through evaporation in water-limited systems. The combination of those two effects will result in higher soil moisture under vegetated soil and lower soil moisture in bare soils. However, this was not universal, a negative relationship was found between soil moisture and canopy cover at low soil moisture and the negative relationship diminished when soil became wetter [43]. More intense temporal soil moisture fluctuations can be found in the top soil layer than in the deeper layers. This result is in good agreement with the observations in Wagga Wagga and Tarrawarra [44] and soil moisture fluctuations may became less pronounced in deeper soil layers as suggested by recent studies in China [45, 46]. At GPG, mean soil moisture and standard deviation in the top layer (1.97% and 0.39% respectively) were more than two times that in the deeper layer (0.62% and 0.19% respectively), whereas the CV of the deeper layer (30.65%) was nearly double that in the top layer (19.79%) (Table 1). The soil moisture difference between top layer and deeper layer in at GPG (bare soil) might be induced by the soil properties. As suggested by previous studies, soils at the gravel plains around GPG are primarily calcrete [33, 36]. A study conducted in semiarid southern New Mexico suggested that caliche can absorb considerable amount of water [47] which is not easy to be released to the surrounding soil or taken up by vegetation. In addition, calcrete soil has low infiltration capacity limiting water movement toward into deeper soil layers. In SDG, there was no significant difference (p > 0.05) in average soil moisture between bare soil (0.57%) and under vegetation cover (0.73%) microsites (Table 1). This is consistent with a previous study which found the average soil moisture is nearly the same in the two microsites (bare soil and covered with vegetation, respectively) along the Kalahari Transect [17]. The soil moisture standard deviations were very similar in both microsites, while their CVs were different with bare soil (20.05%) being two times that of microsites covered with vegetation (10.96%). At GPK, the mean soil moisture (0.70%) was similar to the soil moisture for bare soil at SDG (p > 0.05) (Table 1). This can be clearly reflected by the soil moisture pdfs at GPK and SDG from which nearly the same shapes have been observed (Fig 3). The CV (57.14%) for GPK was the highest when comparing with the microsites at GPG and SDG.

At GPG and GPK, soil moisture always followed the rainfall patterns with soil moisture peaks following rainfall events, whether the surface was covered with vegetation or not (Fig 2). At the top soil layer, positive correlations between rainfall and soil moisture dynamics were more obvious (Table 2). However, soil moisture dynamics did not have any correlation with rainfall at SDG (Table 2). The inconsistencies between the three sites were presumably caused by the infiltration capacity. In our field measurements, higher *K*_s_ (50.6 m day^-1^) (Table 3) was observed at SDG, which was nearly one order of magnitude higher than that of GPG (5.6 m day^-1^) and GPK (3.5 m day^-1^) (Table 3). This indicates that water would leach away at a fast rate at SDG and can hardly be retained by the soil, whereas some water might be captured by soil at GPG and GPK after rainfall events.

**Table 2:** The correlations between rainfall and soil moisture for different layers of gravel plain at Gobabeb (GPG), sand dune at Gobabeb (SDG) and gravel plain at Kleinberg (GPK).

Study site	Bare soil/Vegetation	Depth (cm)	r	P
Gravel plain	***Bare soil***	7.5	0.49	0.00
(Gobabeb)	***Bare soil***	22.5	0.03	0.53
	Vegetation	7.5	0.03	0.52
Sand dune	Bare soil	15	0.07	0.17
(Gobabeb)	Vegetation	7.5	0.01	0.82
Gravel plain	Bare soil	5.0	0.39	0.00
(Kleinberg)				

**Table 3:** Soil, vegetation and rainfall parameters for gravel plain at Gobabeb (GPG), sand dune at Gobabeb (SDG) and gravel plain at Kleinberg (GPK).

	Unit	Gravel plain	Sand dune	Gravel plain
		(Gobabeb)	(Gobabeb)	(Kleinberg)
		Bare soil	Bare soil	Bare soil
Soil parameters				
Porosity	*n*	0.34	0.40	0.48
Field capacity	*s*_fc_	0.10	0.05	0.09
Hygroscopic point	*s*_h_	0.05	0.015	0.01
Saturated hydraulic conductivity	*K*_s_ (m day^-1^)	5.60	50.60	3.50
Soil depth	*Z*_r_ (m)	0.21	0.48	0.35
Rainfall parameters				
Average storm frequency	λ (day^-1^)	0.01	0.01	0.04
Average storm depth	α (mm)	2.62	3.02	1.95
Vegetation parameters				
Maximum evapotranspiration	*E*_max_ (mm day^-1^)	1.25	1.15	2.20
Soil-vegetation parameters				
Point of incipient stress	s*	0.09	0.03	0.08
Permanent wilting point	*s*_w_	0.075	0.020	0.045

### 3.2 Sensitivity analyses

The modeled soil moisture sensitivities to the model parameters from both bounded and non-bounded groups were tested. In general, the model was more sensitive to the parameters in the bounded group with the average sensitivity ranging from 0.00011% to 44%. In comparison, the average sensitivity ranged from -0.00065% to 0.07% for the non-bounded group (Table 4). For parameters in the bounded group, the sensitivities were all positive except *n* at SDG. This indicates that simulated soil moisture will increase as values of these parameters become higher. In the non-bounded group, however, nearly all the parameters had negative sensitivity except *Z*_*r*_ at GPG and GPK.

**Table 4:** Model sensitivity of the key parameters for gravel plain at Gobabeb (GPG), sand dune at Gobabeb (SDG) and gravel plain at Kleinberg (GPK).

	Unit	Step	Gravel plain (Gobabeb)		Gravel plain (Kleinberg)		Sand dune (Gobabeb)	
			Interval	Average sensitivity(%)	Interval	Average sensitivity(%)	Interval	Average sensitivity(%)
Bounded								
Porosity	*n*	0.001	[0.002, 0.981]	**0.75**	[0.006, 0.969]	**0.00011**	[0.082, 0.899]	**-0.31**
Field capacity	*s*_fc_	0.001	[0.001, 0.156]	**15**	[0.001, 0.585]	**1.4**	[0.001, 0.04]	**1.9**
Hygroscopic point	*s*_h_	0.001	[0.001, 0.094]	**44**	[0.001, 0.404]	**11**	[0.001, 0.059]	**2.1**
Point of incipient stress	s*	0.001	[0.001, 0.985]	**0.75**	[0.001, 0.847]	**0.17**	[0.001, 0.984]	**0.37**
Permanent wilting point	*s*_w_	0.001	[0.001, 1]	**0.086**	[0.001, 1]	**2.6**	[0.001, 0.964]	**3.2**
Non-bounded								
Soil depth	*Z*_r_ (m)	0.01	[0.01, 15.50]	**0.07**	[0.06, 15.83]	**0.032**	[0.1, 18.11]	**-0.036**
Maximum transpiration	T_max_ (mm day^-1^)	0.01	[0.01, 11.04]	**-0.11**	[0.01, 11.13]	**-0.036**	[0.01, 7.77]	**-0.06**
Maximum evaporation	E_w_ (mm day^-^1)	0.01	[0.01, 13.75]	**-0.11**	[0.01, 8.36]	**-0.0014**	[0.01, 4.27]	**-0.057**
Saturated hydraulic conductivity	*K*_s_ (m day^-1^)	0.1	[0.1, 81.4]	**-0.00065**	[0.1, 82.6]	**-0.0044**	[0.1, 100]	**-0.00041**

Sensitivity analyses suggested that parameter sensitivities depended on the overall model parameterization, thus the parameter sensitivity was different for each site. For the bounded group at GPG, average sensitivities of *s*_*h*_, *s*_*fc*_, *n* and s* were of the same order of magnitude. Among the bounded group, *s*_*h*_ had the largest average sensitivity thousands of times more than the average sensitivity of *s*_*w*_. In the non-bounded group, the vegetation parameter *T*_*max*_ and *E*_*w*_ exhibited the same sensitivity value of -0.11% while average sensitivity values of soil parameter *Z*_*r*_ and *K*_*s*_ were quite different, with 0.07% for *Z*_*r*_ and -0.00065% for *K*_*s*_ (Table 4). For all parameters at GPK, *s*_*h*_ was the most influential factor for the model among all the parameters with an average sensitivity value of 15%. In the bounded group, the minimum average sensitivity was *n*, which was thousands of times smaller than that at GPG. In the non-bounded group, all the average sensitivity values were negative except *Z*_*r*_, which was far larger than any other values of the group. With respect to SDG, *s*_*h*_ had the largest average sensitivity value and it had the same magnitude with *s*_fc,_ which was very similar to that at GPG (Table 4). But the average sensitivity of *n* had a totally distinct trend to that of the other two sites suggesting that simulated soil moisture will decrease as *n* goes higher within the predefined interval. The same patterns happened in the non-bounded group in which the average sensitivity of *Z*_*r*_ was -0.036% (Table 4), though the absolute value of *Z*_*r*_ had the same magnitude as those at the other two sites. For average sensitivities of the three sites, the model was more sensitive to *s*_h_ and *s*_fc_ and less sensitive to *K*_s_. All the soil parameters had positive values except *n*, *Z*_*r*_ of SDG and *K*_s_ of all the sites whereas all the vegetation parameters had negative values.

### 3.3 Stochastic modeling of soil moisture dynamics

**Fig 4:**
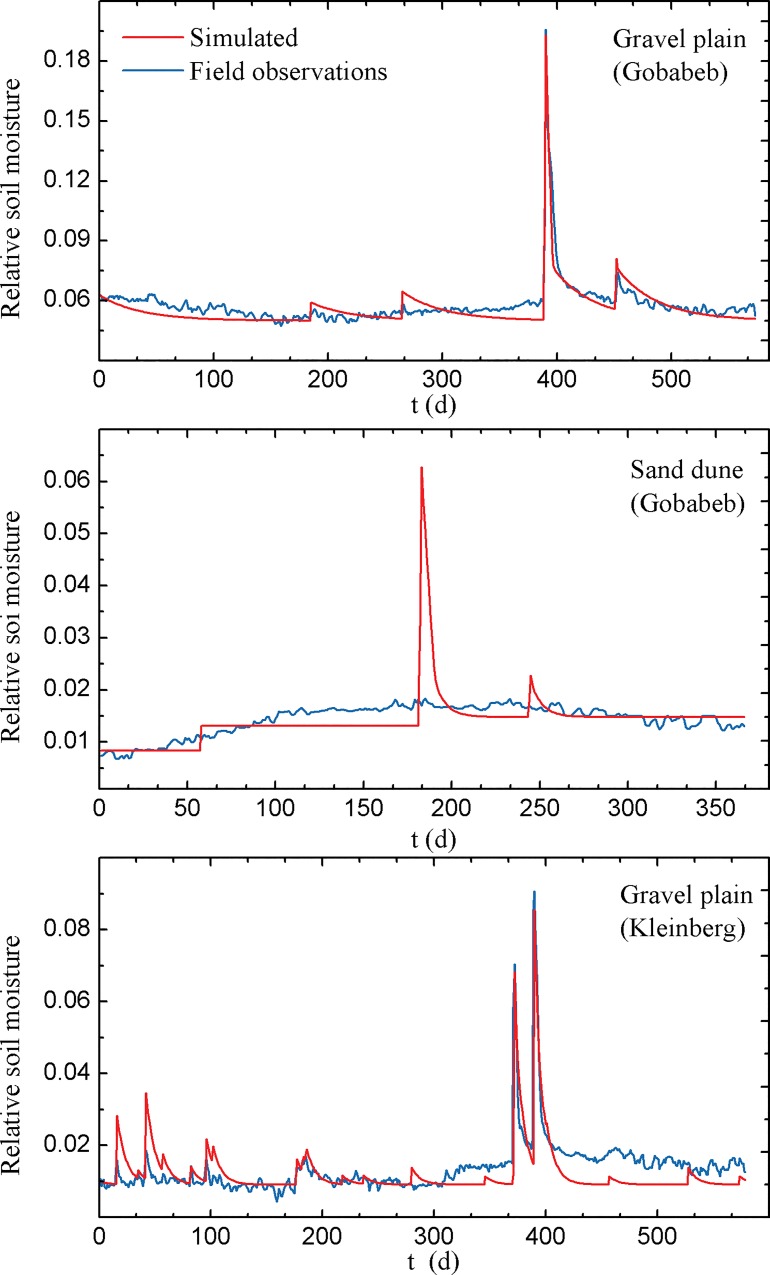
The comparison between field observations and simulated relative soil moisture patterns in gravel plain at Gobabeb (GPG), sand dune at Gobabeb (SDG) and gravel plain at Kleinberg (GPK).

Soil moisture from the three sites (7.5 cm bare soil at GPG; 15 cm bare soil at SDG and 5 cm at GPK) were selected and simulated by a stochastic modeling framework. The modeled mean relative soil moisture and soil moisture dynamics in general agreed well with field observations (Fig 4).

Simulated mean relative soil moisture at the three sites was 0.056 for GPG, 0.014 for SDG and 0.015 for GPK (Table 5) and mean soil moisture observed in the field was 0.058, 0.014 and 0.015 for GPG, SDG and GPK, respectively (Table 5). Soil moisture at GPG can be well simulated, with soil moisture peaks corresponding to rainfall regimes. Fig 4 shows that the simulated GPG soil moisture was slightly lower than soil moisture acquired from the field, with soil moisture increasing sharply to the peak during a rainfall event, and decreasing rapidly to a soil moisture baseline right after a rainfall event. The sharp decrease of soil moisture might be explained by the modeling assumption that rainfall is the only contributor to the increase of soil moisture. When intense rainfall inputs were added, soil moisture would immediately increase to field capacity and leakage would instantaneously occur until soil moisture returned below field capacity. For SDG, intense soil moisture peaks can be observed from simulated results, with each soil moisture peak directly following the rainfall patterns (Fig 4). Although rainfall patterns at SDG are the same as that in GPG, the soil moisture simulation results at SDG were quite different. Soil moisture of SDG suddenly increased to a fixed value when a storm came and stayed at that value until another rainfall came which is different from the persistent decrease of soil moisture at GPG. This is because our initial soil moisture at SDG was below *s*_*h*_ and the first rainfall may not have been sufficient for soil to reach its hygroscopic point. This caused a flat and a sharp increase of soil moisture in the beginning. On the other hand, simulated soil moisture of SDG always reached the baseline soil moisture without any smooth soil moisture transition or soil moisture fluctuation. In contrast, the soil moisture curve of GPG reached the baseline value very smoothly, though still without any soil moisture fluctuation (Fig 4). The differences may be induced by soil properties where larger *K*_s_ and smaller *s*_fc_ was found in SDG (Table 3). For GPK, fluctuations can be seen when rainfall came. At the beginning, simulated soil moisture was slightly higher than the measured soil moisture value but after two intense rainfall events the simulated results were smaller than field observations (Fig 4). In general, soil moisture patterns and mean relative soil moisture can be well-simulated by the stochastic model. However, daily soil moisture fluctuations cannot be fully revealed by the model simulation. From our perspective, two factors mainly influence the model output. The first factor is the effect of non-rainfall components, particularly fog and dew, which influence the daily soil moisture fluctuations while the model failed to take them into account resulting in steep slopes of soil moisture of two adjacent days between and after a rainfall event. It has been suggested that fog has been persistent in Namib Desert [48]. High fog is the most common type of fog that can be found at Gobabeb, which arrives during early morning hours and dissipates quickly with sunrise when the surface temperature rises. This is probably the first reason why we cannot simulate the daily soil moisture fluctuations. In addition to the non-rainfall component effect, the modeling framework itself is based on the assumption that daily scale infiltration and redistribution occur instantaneously, and soil moisture that exceeds field capacity will be drained away immediately [49]. Moreover, the model does not consider vertical distribution of soil moisture, assuming soil moisture is the same along soil columns. In reality, however, soil moisture is different from one layer to another, with soil moisture in a shallow layer generally having higher soil water content than that in a deep layer.

**Table 5:** The observed and simulated relative soil moisture (mean ± standard deviation) for gravel plain at Gobabeb (GPG), sand dune at Gobabeb (SDG) and gravel plain at Kleinberg (GPK).

Study sites	Gravel plain	Sand dune	Gravel plain
	Gobabeb	Gobabeb	Kleinberg
Soil type	Bare soil	Bare soil	Bare soil
Depth (cm)	7.5	15	5.0
Observed	0.058±0.010	0.014±0.003	0.015±0.008

Simulated	0.056±0.011	0.014±0.006	0.015±0.008


Our simulation results showed that the stochastic model can be used to simulate soil moisture patterns in the Namib Desert especially in gravel plains where finer soil texture was found. In the site with coarse soil texture (e.g., SDG), the model did not perform very well, indicating that the modeling framework may not be able to accurately predict soil moisture dynamics at daily scale for sites with coarse texture.

## 4. Summary

In this study, twelve to nineteen months’ daily-scale soil moisture and rainfall data were obtained from three sites located within the Namib Desert. The ground observations showed that soil moisture was controlled by rainfall patterns at GPG and GPK, particularly for shallow soil layers with strong correlations between soil moisture and rainfall, while weak rainfall-soil moisture correlation was found at the sand dune site. The field observations were simulated using a process-based modeling framework. The modeled soil moisture patterns and mean soil moisture values agreed well with field observations. However, soil moisture fluctuations cannot be simulated and require future work such as taking fog and dew into consideration as additional water inputs. The model sensitivity showed that sensitivity patterns were quite similar between the three sites. But the sensitivity magnitude of the model parameters differed from each other, with *s*_h_ and *s*_fc_ having the largest sensitivities among all the parameters. The sensitivity analyses of the three sites were quantified and can be used as an uncertainty indicator for this modeling framework in future applications.

## Supporting Information

S1 Table The daily rainfall and soil moisture data from Gravel plain at Gobabeb (January 2, 2014 to July 28, 2015), Sand dune at Gobabeb (July 28, 2014 to July 28, 2015) and Gravel plain at Kleinberg (January 1, 2014 to August 3, 2015). (XLSX)
